# 17th Annual Report

**Published:** 1891-07

**Authors:** 


### REVIEWS.

17th ANNUAL REPORT OF THE SECRETARY OF THE STATE BOARD OF HEALTH, of the STATE OF MICHIGAN, for the fiscal year ending June 30, 1889. By authority. Lansing, Michigan: Darius D. Thorp, Printer 1890. 324 pages, contains a theory, page xxxvii, that Typhoid Fever may be intercommunicable between men and animals. The Board ot Health has authorized experiments in this direction; reports a case of death of a man from glanders, and other cases of glanders throughout the State. *Supplement.* Proceedings and addresses at a Sanitary Convention, held at Alpena, Michigan.

HYPODERMIC MEDICATION (Manual of). By G. Archie Stockwell, M.D., F. Z. S. George S. Davis, Detroit, Michigan, 1890. This little book of 158 pages, gives a history of Hypodermic Medication, the method of preparing solutions for injections, the instruments used, and a manual of subcutaneous injections, which gives the principal drugs used hypodermically, the strength of solution to be used, the size of dose, and the physiological action it produces.

AN EPITOME OF THE NEWER MATERIA MEDICA. Parke, Davis & Co., Detroit, Michigan, 1890. This is a short appendix to the Materia Medica, containing a list of the drugs, and the preparations of them, recently brought into use.

